# Correlation of short knee and full-length X-rays in evaluating coronal plane alignment in total knee arthroplasty

**DOI:** 10.1186/s13018-022-03246-7

**Published:** 2022-08-08

**Authors:** Seyyed-Morteza Kazemi, Seyyed-Mohammad Qoreishi, Arash Maleki, Reza Minaei-Noshahr, Seyyed-Mohsen Hosseininejad

**Affiliations:** 1 Bone Joint and Related Tissues Research Center, Akhtar Orthopedic Hospital, Shahid Beheshti University of Medical Sciences, Tehran, Iran; 2 Clinical Research and Development Unit, Akhtar Orthopedic Hospital, Shahid Beheshti University of Medial Science, Tehran, Iran; 3 Joint, Bone, Connective Tissue Rheumatology Research Center (JBCRC), Golestan University of Medical Sciences, Gorgān, Iran

**Keywords:** Total knee arthroplasty, Short X-rays, Full length X-rays, Mechanical alignment, Anatomical alignment

## Abstract

**Background:**

Coronal alignment after total knee arthroplasty (TKA) would influence the implant survival. Coronal alignment could be measured on short and full-length X-rays. The goal of the current study was to assess the correlation of short and full-length X-rays to accurate prediction of the true Hip-Knee-Ankle alignment after TKA in the Iranian population.

**Methods:**

Lateral distal femoral and medial proximal tibial angles, FTA, HKA, in 180 Iranian patients (243 knees without extra-articular deformities) were measured and compared on short and full-length standing X-rays of primary TKA pre/postoperatively.

**Results:**

The correlation between the preoperative FTA-short and FTA-long, FTA-short and HKA, and FTA-long and HKA values in degrees were fair, good and good (*r* = 0.64) (*r* = 0.73), (*r* = 0.76), respectively. This correlation for postoperative aMPTA and mMPTA (*r* = 0.73), and FTA-short and HKA (*r* = 0.76) values were good and significant (*P* = 0.001). Also, assessing coronal alignment based on short and full-length measurements would result in varying pre/postoperative alignments (varus, neutral and valgus).

**Conclusion:**

Full length X-rays could not be replaced by short knee X-rays to asses true coronal alignment in TKA; considerable portion of our cases were missorted as varus, neutral or valgus based on the FTA versus the HKA. Intraoperative fixed 5° valgus angle cut of distal femur did not result in postoperative favorable neutral alignment in all cases.

**Level of evidence:**

IV.

## Introduction

Postoperative coronal plane limb alignment is an imperative issue in increasing total knee arthroplasty (TKA) survivability [1–5]. The full-length hip-knee-ankle angle (HKA) on mechanical axis as a standing anteroposterior [6, 7] radiographs, and the femorotibial angle (FTA) on anatomical axis as a short standing AP knee radiographs, could be used to measure the limbs coronal alignment [4, 8–11]. The latter requires less complex technique and equipment, incurs fewer costs, and also exposes the other organs to less radiation. As a result, most surgeons prefer anatomical FTA measured from short knee AP radiographs to determine post-TKAs alignment [3, 4, 7, 12].

Furthermore, extra-articular deformities including femur or tibial bowing have been identified to potentially affect the aforementioned axes [7, 13, 14]. There have been some previous reports on TKA alignment indicating minor discrepancies with high correlations between the short knee and full-length radiographies (SKR, FLR), in addition to some other reports indicating larger differences and poor correlations between SKR and FLR radiographs; it is debatable whether FLR could substitute for SKR [6, 13, 15–17].

It is important to use an accurate and practical protocol for pre/post-TKA X-rays in spite of minimum exposure to radiation and improving costs efficacy.

SKRs could potentially save institutions money, be easier to access, and reduce radiation exposure to patients [7].

We hypothesized that SKR could be used instead of FLR to assess lower limb coronal alignment in TKA. The hypothesis can be established if the SKR and FLR measurements are strongly related.

In the literature, there is an empirical "confidence interval" for distal femur cut with a valgus angle range of 4–7° [18–21]. By default, all cases in our orthopedic surgery department are prepared to insert the femoral component at a 5-degree valgus angle.

We also assumed that in cases without extra-articular deformities, the default intra-operative 5 valgus angle would be sufficient to maintain appropriate coronal alignment; if the post-operative specified measurements were within acceptable ranges (MPTA and LDFA: 88-92), the hypothesis would be feasible.

In the current study, we attempted to conduct a comparison of the alignment analysis between knees without extra-articular deformities to provide an applicable assessment on whether SKR could be appropriate enough to establish acceptable coronal plane alignment after TKA in Iranian cases; we also intended to evaluate the post-operative alignment measurements, especially MPTA and LDFA, after applying an empirical distal femur valgus cut of 5°.

## Materials and methods

After receiving Local Ethical board approval, 243 knees from 180 Iranian patients undergoing TKA procedure by the same surgeon from October 2016 to October 2020 were retrospectively investigated in a cross-sectional study.

The exclusion criteria included congenital limbs anomalies, ipsilateral limb post-traumatic fractures, neuromuscular disorders, rheumatoid/traumatic arthritis, osteonecrosis, more than 10° flexion contracture, history of operation on the ipsilateral limb, inadequate rotation on radiographs, and ambulatory and/or standing discomforts.

Weight-bearing FLR were obtained with the patients bare foot and the knees in extension in a position as the patella was oriented direct anterior. Deformities were defined as tibial and femoral bowing as an FBA > 3°, laterally or medially [17, 22, 23].

Preoperatively, the tibial and femoral bowing angles (TBA, FBA) were assessed on standard AP FLR X-rays to exclude the knees with extra-articular deformities. Postoperatively, routine knee weight-bearing AP and lateral X-rays and FLR were obtained at 6 weeks. Anatomical MPTA, anatomical LDFA and FTA-short were measured on short AP knee X-rays. The mechanical MPTA, mechanical LDFA, FTA-long and HKA were also measured on FLRs.

coronal limb alignment was categorized into varus, neutral and valgus based on an FTA-short values < 2.4°, 2.4° to 7.2°, and > 7.2°, respectively; in full-length X-rays, the varus, neutral and valgus values would be <  − 3°, − 3° to 3°, and > 3° based on HKA measures, correspondingly. Moreover, for the tibial component alignment after arthroplasty, aMPTA value below 88° was graded as varus, 88° to 92° as neutral, and above 92° as valgus[4, 8, 9].

Intra-operatively, the valgus angle of 5° for distal femoral cut was made using conventional intramedullary femoral Jig based instrumentation in all cases and the coronal alignment was measured on AP standing HKA X-ray.

The same observers conducted the radiographic measurements to eliminate intra-observer errors. All data were imported into and categorized in Microsoft Excel (Microsoft Corporation) and computed by SPSS for Windows version 16.0 (SPSS, Chicago, IL).

Correlation coefficients were classified by formerly defined semiquantitative values: excellent for 0.9 ≤ *r* ≤ 1, good for 0.7 ≤ *r* ≤ 0.89, fair/moderate for 0.5 ≤ *r* ≤ 0.69, low for 0.25 ≤ *r* ≤ 0.49, and poor for ≤ 0.24 [24]. *P* values less than 0.05 were considered as significant results.

## Result

Two-hundred forty-three radiographies of 180 Persian participants were investigated. The patients’ mean ± SD (range) age was 67.16 ± 6.73 (56–82). Of 180, Male patients made up 45 (25%) of the limbs, the rests were female (135 (75%)). The mean ± SD (range) for patients’ BMI was 29.46 ± 2.18 [21–32].

The correlation coefficient for the preoperative FTA-short and FTA-long, FTA-short and HKA, and FTA-long and HKA values in degrees were fair, good and good (*r* = 0.64), (*r* = 0.73), (*r* = 0.76), respectively. The correlation coefficient for preoperative aMPTA and mMPTA was low (*r* = 0.29), this value between aLDFA and MLDFA was also low (*r* = 0.42) (Table 1).

**Table 1:** Preoperative Correlations and their associated *P* values between measurements on Short and Full-Length lower limb radiographies

	MPTA/mMPTA	aLDFA/mLDFA	FTA-short/HKA	FTA-short/FTA-long	HKA/FTA-long
*r*	0.29	0.42	0.73	0.64	0.76
*P*	0.19	0.055	0.001	0.006	0.001

Underline indicates the significance level is *P* < 0.05

The correlation coefficient between postoperative aMPTA and mMPTA, and FTA-short and HKA values were both good (*r* = 0.71). The correlation coefficient between the FTA-short and FTA-long, and FTA-long and HKA values were (*r* = 0.29), (*r* = 0.23), respectively; this value between aLDFA and MLDFA was also low (*r* = 0.4) (Table 2).

**Table 2:** Postoperative Correlations (*r*) and their associated *P* values between values on Short and Full-length lower limb X-rays

	aMPTA/mMPTA)	aLDFA/mLDFA	FTA-short/HKA	FTA-short/FTA-long	HKA/FTA-long
*r*	− 0.71	0.4	− 0.71	0.29	0.23
*P*	0.80	0.90	0.80	0.32	0.27

The mean postoperative mMPTA and mLDFA were 88.08 and 90.73 degrees, respectively. The mean value for postoperative HKA and FTA-long were − 0.18, 3.07, correspondingly (Table 3).

**Table 3:** Pre/Postoperative mean ± SDs for anatomical and mechanical values

Short knee X-ray				Full length X-ray			
	Short a-FTA	aMPTA	aLDFA	HKA	mMPTA	mLDFA	FTA-long
Preoperative	− 5.45 ± 7.54	81.71 ± 3.57	85.41 ± 3.51	− 17.42 ± 14.29	79.1 ± 4	94.1 ± 5.1	− 26.21 ± 59.5
Postoperative	− 1.25 ± 17.31	88.25 ± 2.19	85.33 ± 2.1	− 0.18 ± 7.24	88.08 ± 2.49	90.73 ± 3.74	3.07 ± 3.25

After TKA, 53.8% of patients had a neutral alignment on postoperative short knee X-rays while only 28.6 percent had neutral alignment on full-length films.

The alignment categories (varus, neutral, and valgus) based on short and full-length measurements has been given in detail in Table 4.

**Table 4:** Varus/Neutral/Valgus alignment based on measurement on short and full-length X-rays

	Varus %	Neutral %	Valgus %
Preoperative FTA-short	81.5	9.5	9.5
Preoperative FTA-long	70.6	23.5	5.9
Preoperative HKA	88.2	0	11.8
Postoperative FTA-short	46.2	53.8	0
Postoperative FTA-long	35.7	35.7	28.6
Postoperative HKA	57.1	28.6	14.3

## Discussion

The majority of surgeons continue to reach a neutral mechanical alignment in TKA [25]. Our findings showed that TKAs seeming to have neutral alignment on short knee X-rays were in varus or valgus on full-length standing X-rays based on HKA. Adequate evaluation of pre/postoperatively coronal lower limbs alignment is thought to be important in TKA [26–28]. Furthermore, the longevity of a TKA is heavily related to maintaining normal alignment, as several reports in the literature have indicated that misalignment of a TKA raises contact stresses across the implant and could be one of the causes of failure [29–31]. However, recent studies have questioned the theory that a neutral mechanical alignment is correlated with improved implant survival [3, 4, 8, 9]. Parratte et al. [8] found no difference in a 15-year survival TKA with a postoperative HKA of 0° to 3°. Bellemans et al. [10] and Vanlommel et al. [32] reported that cases with varus alignment preoperatively had better clinical and functional conditions if they had had mild preoperative varus (HKA = 3° to 6° of varus) rather than neutral (HKA = 0° to 3° of varus) or extreme varus (HKA > 6° varus). While some authors [4, 9] linked survival to the FTA-short, survival and functional performance was linked to the HKA in other reports [8, 10, 32]. This discrepancy has made it difficult to compare results from one study to another, as well as to apply study findings to patients who do not have the corresponding type of postoperative radiograph.

Several studies have been conducted to examine the correlation between coronal alignment measurements on short and long X-rays. Van Raaij et al. [13] examined the FTA and HKA and discovered a mild correlation (*r* = 0.65); Kraus et al. [33] revealed the same association (*r* = 0.65) for the FTA and HKA. Hinman et al. [16] found a higher correlation (*r* = 0.88) for FTA and HKA, while Issa et al. [6] detected a similar correlation (*r* = 0.86) for FTA and HKA. Though, the last two mentioned studies applied conventional short knee X-rays-Hinman et al. calculated the HKA and the FTA on the same full-length X-rays, and Issa et al. placed the knees in semi-flexion for the short knee X-ray rather than the usual extended position [6, 16].

There have been few studies that compare short and long X-rays of TKA postoperatively. Petersen and Engh [34] compared the FTA-long with short and discovered a 1.4° difference, but did not test the HKA. Likewise, Ishii et al. [35] collated postoperative anatomical axis on short and long X-rays but not mechanical axis alignment. A latest report by Abu-Rajab et al. [14] found poor consistency (= 0.07) between the mechanical axis on short and long X-rays. They did not, however, collate the anatomical alignment on the short X-ray with the mechanical alignment on the full-length X-ray; rather, they collated the HKA to the mechanical axis on the short X-ray, that they described as the angle between the base of the tibial component and the tibial anatomical axis; such approach disregards involvement of the femur in total limb alignment and is not consistent with the assessment of coronal plane alignment in studies documenting clinical outcomes of TKA based on short X-rays [4, 12, 14].

Alzahrani et al. [27], like Kraus et al. [33], demonstrated that short knee X-rays had excellent to good association with the full-length X-rays in widely applied alignment measures in TKA (MPTA, FTA and LDFA) pre and postoperatively.

There are some possible drawbacks to this analysis that must be addressed when interpreting the findings. Lower extremity rotation will influence our measurements, as it does in all radiographic assessments of coronal plane alignment. However, all of our radiographs were collected in accordance with a single official procedure for all cases, which may have mitigated this possible limitation. We did not test inter- and intra-observer reliability of our data, however previous researches has shown that the indicators used in our current study have significant inter- and intra-observer reliability [7, 36].

There is still room for debate whether choosing a fixed angle valgus cut (5–7°) of distal femur or applying individualized valgus cut for each patient would be suitable to achieve standard post-operative knee alignment; as there are some reports supporting personalized cutting techniques. Conversely, other reports have noted that fixed angle cuts of distal femur results in favorite alignment in most cases [37–41]; it seems that the simplified fixed angle (e.g., 5°) technique suits for uncomplicated cases once individualized equipment are not accessible, given that final angle cut of distal femur may not affect patients’ functional short-term outcomes[21, 41].

## Conclusion

As we figured out in this study, SKR and FLR are not fully correlated in Persian population; short knee radiographies are inadequate in evaluating coronal alignment of TKA candidates. A Considerable portion of cases were missorted as varus, neutral or valgus based on the FTA versus the HKA.

Intra-operative valgus cut of 5° for distal femur in spite of any preoperative alignment angle values in TKA patients without extra-articular bone deformities would not totally result in postoperative neutral full-lengths alignments in our Persian population.

## Data Availability

Not applicable.

## Abbreviations

TKA: Total knee arthroplasty
HKA: Hip-knee-ankle
LDFA: Lateral distal femoral angle
a/mMPTA: Anatomical/mechanical medial proximal tibial angles
FTA: Femoral tibial angle
SKR: Short knee radiography
FLR: Full-length radiography
